# Using models to shape measles control and elimination strategies in low- and middle-income countries: A review of recent applications

**DOI:** 10.1016/j.vaccine.2019.11.020

**Published:** 2020-01-29

**Authors:** F.T. Cutts, E. Dansereau, M.J. Ferrari, M. Hanson, K.A. McCarthy, C.J.E. Metcalf, S. Takahashi, A.J. Tatem, N. Thakkar, S. Truelove, E. Utazi, A. Wesolowski, A.K. Winter

**Affiliations:** a Department of Infectious Disease Epidemiology, London School of Hygiene and Tropical Medicine, London, UK; b Vaccine Delivery, Global Development, The Bill & Melinda Gates Foundation, Seattle, WA, USA; c Center for Infectious Disease Dynamics, Pennsylvania State University, University Park, PA, USA; d Institute for Disease Modeling, 3150 139th Ave SE, Bellevue, WA 98005, USA; e Department of Ecology and Evolutionary Biology, Princeton University, Princeton, NJ, USA; f Department of Medicine, University of California San Francisco, San Francisco, CA 94143, USA; g WorldPop, Department of Geography and Environmental Science, University of Southampton, Highfield, Southampton SO17 1BJ, UK; h Department of Epidemiology, Bloomberg School of Public Health, Johns Hopkins University, Baltimore, MD, USA

**Keywords:** Measles, Measles vaccination, Rubella, Epidemiology, Elimination, Mathematical models

## Abstract

After many decades of vaccination, measles epidemiology varies greatly between and within countries. National immunization programs are therefore encouraged to conduct regular situation analyses and to leverage models to adapt interventions to local needs. Here, we review applications of models to develop locally tailored interventions to support control and elimination efforts. In general, statistical and semi-mechanistic transmission models can be used to synthesize information from vaccination coverage, measles incidence, demographic, and/or serological data, offering a means to estimate the spatial and age-specific distribution of measles susceptibility. These estimates complete the picture provided by vaccination coverage alone, by accounting for natural immunity. Dynamic transmission models can then be used to evaluate the relative impact of candidate interventions for measles control and elimination and the expected future epidemiology. In most countries, models predict substantial numbers of susceptible individuals outside the age range of routine vaccination, which affects outbreak risk and necessitates additional intervention to achieve elimination. More effective use of models to inform both vaccination program planning and evaluation requires the development of training to enhance broader understanding of models and where feasible, building capacity for modelling in-country, pipelines for rapid evaluation of model predictions using surveillance data, and clear protocols for incorporating model results into decision-making.

## 1 Introduction

The Global Vaccine Action Plan 2011–2020 (GVAP) has a target of eliminating measles and rubella in at least five of the six World Health Organization (WHO) regions by 2020 [1]. The WHO recommends that all countries include a second dose of measles-containing vaccine (MCV) in national vaccination schedules; countries aiming for measles elimination should achieve ≥95% coverage with both doses equitably across all districts. To attain these goals, routine immunization (RI), the regular vaccination of children according to the national immunization schedule, may be complemented by supplementary immunization activities (SIAs), short duration national or subnational campaigns targeting all children within a chosen age range [2]. Progress is very heterogenous [3] hence current measles epidemiological profiles range from persistent high incidence and mortality in some, mainly low- and middle-income countries (LMICS), to sporadic outbreaks, to the elimination of indigenous transmission [4]. Models have been used to analyse measles dynamics since the 1920s and have informed global public health policy [2], [5]. The WHO and the Vaccine Alliance (Gavi) now recommend that countries use models when possible to adapt vaccination strategies to local disease dynamics [2], [6].

**Fig. 1:**
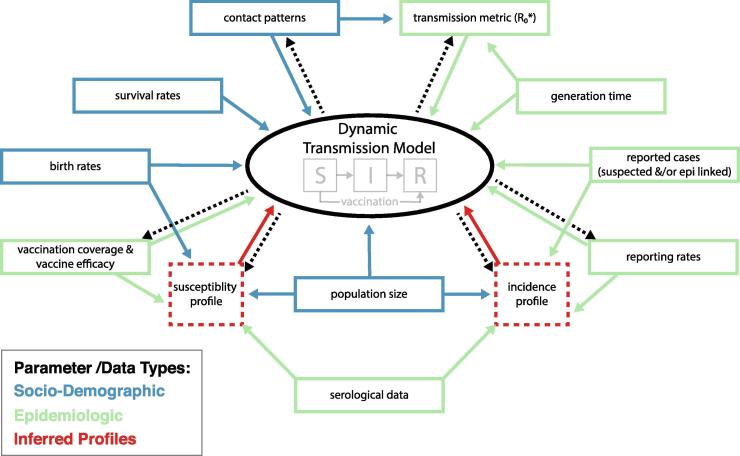
Schematic of inputs (solid lines) and outputs (dashed lines) from a Susceptible [S] – Infected [I] – Recovered [R] dynamic transmission model for measles and rubella. The colors represent different types of parameters/data: socio-demographic are blue, epidemiologic are green, and red represents inferred epidemiologic profiles. The model can be structured by time, age, or space, or any combination of these dimensions. *R_0_ or the basic reproduction number is the average number of people a typical case will infect in a completely susceptible population. Estimates for the R_0_ of rubella range from 2 to 12, with an estimated median of just over 5 [48], and the measles R_0_ is typically reported between 12 and 18, although can range from 1 to 770 [42]. (For interpretation of the references to colour in this figure legend, the reader is referred to the web version of this article.)

In this paper, we review how models are increasingly used to address programmatic questions at the country level in LMICS (Table 1). We illustrate how combining different sources of data in models helps to overcome some of the biases in individual data streams. Although there are many different types of models, we highlight some of the more common forms and their applications. We begin with examples of statistical and semi-mechanistic transmission models, which are used in conjunction with demographic data to estimate the spatial and age-specific distribution of vaccination (from coverage data) and infection (from surveillance data). From these, the location, size and ages of the susceptible population can be estimated [7]. These results are useful in themselves and are input into dynamic transmission models, where the spread of infection is explicitly modelled to estimate how likely transmission is in different population groups, estimate the magnitude of outbreaks, and assess the impact of various vaccination strategies to guide outbreak response or elimination policies (Fig. 1). In turn, modelling helps to show priorities for the collection of further empirical data on infection and immunity [8]. We focus on the contribution of models to measles control and elimination strategies but point out areas of concern for rubella. Other types and uses of models at the global level, such as disease burden estimation [9] and supply chain alternatives [10], are beyond the scope of this paper.

**Table 1:** The potential contribution of models to answer program questions.

Program question	Empirical data sources	Main disadvantages of current analyses of empirical data	Contribution of models to data analysis and interpretation
Where are gaps in routine MCV coverage?	– Routine reports on vaccinations administered compared to estimated target population, from district level up – Vaccination coverage or multipurpose household surveys (e.g. Demographic and Health Survey (DHS), Multiple Indicator Cluster Survey (MICS)), often stratified at provincial level	– Inaccurate denominators (e.g., old census data, seasonality of denominators) – Inaccurate numerators (vaccination outside resident district; reporting biases) – Vaccination surveys usually report data only at the scale at which the survey was stratified (national +/- provincial) – Long delay from survey implementation to data availability – Quality and representativeness of surveys varies; cannot assume high accuracy	Geospatial models can help to improve denominator estimates. Model-based geostatistical analyses of household survey data (e.g. DHS) allow high-resolution vaccination coverage estimation (e.g. 1x1km) with corresponding uncertainty levels. Models can incorporate data on spatial covariates (urbanicity, remoteness, poverty, etc.) to produce more granular coverage estimates. Models can thus estimate coverage at much smaller scale, without following administrative boundaries within or between countries. These gaps would be missed when examining data at a larger (e.g., district) scale. This allows for identifying coverage gaps that are contiguous across borders which highlights the need for coordinated action. Note: Models do not completely overcome all potential biases in the original surveys from which data are taken, although they can incorporate data from other sources to try to compensate. The uncertainty around high resolution estimates may also be difficult to convey succinctly to end-users.

Where are there most unvaccinated persons?	- Vaccination coverage data - Birth cohort data	– As above. – Cohort analyses not frequently performed to estimate cumulative number of unvaccinated persons over time.	Through integration of high-resolution coverage estimates with gridded population data, numbers unvaccinated can be estimated by time and space. This can provide an understanding of the absolute magnitude of risk and allows for more impactful targeting of resources

Where are the remaining persons susceptible to measles?	- Administrative reports of vaccination coverage at district level, used to rank districts in terms of reported coverage.	– Biases in administrative coverage estimates (as above) – Data on natural immunity very difficult to incorporate without modelling	Models can incorporate historical data on routine and SIA coverage (from highest quality available sources) and estimated incidence over time (adjusting for surveillance quality) to predict residual susceptibility at fine scale. Maps showing hot spots of susceptibility can be produced. These estimates can be combined with data on birth rates at the appropriate scale to estimate the size of the annual susceptible birth cohort in each area.

What is the likelihood of transmission in susceptible populations?	- WHO Measles Programmatic Risk Assessment Tool [115] uses data on routine and SIA coverage, incidence in adjacent areas, population density and presence of vulnerable groups to rank districts according to outbreak risk	– Administrative coverage data often only available source at district level – Surveillance data not adjusted for under-reporting or laboratory confirmation – Cannot estimate risk at smaller population levels – Connectivity between *meta*-populations ignored	Dynamic transmission models can estimate transmission at multiple levels of the country down to a small scale. They can incorporate data on the size of the susceptible population, rate of replenishment via (births-MCV coverage), connectivity and migration among meta-populations, age-specific contact patterns and seasonal changes in demography and behaviour, to estimate effective reproduction rate R.

What is the relative importance of the 1st and 2nd dose of MCV in routine programs with or without SIAs?	- Coverage of each dose reported via administrative records. - Periodic surveys	– May be confusion in reporting of a late MCV1 dose and a true 2nd dose, hence unclear exactly what coverage data are showing. – Home based records of SIA doses not always available in surveys and maternal recall less reliable for multi-dose series	Models help illustrate the importance of distinguishing between a late 1st dose and a true second dose, and the different effects of each on population immunity. Models can be used to project the impact of adding MCV2 to a vaccination schedule, under assumptions of the degree of correlation between doses and the distribution of ages at receipt of each dose

How can SIA effectiveness be measured?			
• Coverage of target population	– As above, plus post-campaign coverage surveys (PCCS), which are now required after Gavi-funded SIAs	– Often rely on administrative reports. – Vaccination surveys usually report data only at the scale at which the survey was stratified (national ± provincial)	Conduct geospatial analyses of PCCS data as above, to identify pockets missed by SIA, especially those with high numbers of never-vaccinated children. Use estimates of zero-dose children from the PCCS to calibrate models of routine vaccination and better estimate the remaining never vaccinated children
• Reaching unvaccinated children	– PCCS now include specific question about prior vaccination	– Accuracy of recall of all prior doses unknown and may be poor for older children exposed to multiple prior SIAs. – If high quality PCCS not done, quality of recall of receipt of SIA doses in later surveys (e.g. DHS) unknown	As above. If no PCCS, use other available data (e.g. DHS data) to compare coverage of Diphtheria, Tetanus, Pertussis (DTP)-containing vaccines with that of MCV1 at fine spatial scale to identify areas where MCV1 substantially higher than DTP, which may reflect ability of SIAs to reach those missed by routine services (though other explanations cannot be excluded)
• Reaching previously vaccinated children with a second dose	– As above using PCCS	– As above	Use geospatial models to produce high resolution estimates of coverage with at least 2 doses of MCV and show areas where the SIA has conferred extra protection
• Immunizing susceptible children	– In some small research settings, pre- and post-SIA sero-surveys	– Usually empirical data on this are not available	Use serological data if available, or cohort analysis of coverage and surveillance data, to estimate SIA contribution to immunity. Estimate the reduction in population susceptibility due to the SIA by comparing the goodness-of-fit of calibrated transmission models, which assume a particular efficacy, to data on laboratory confirmed cases (scaled according to the reporting rate)
• Reducing transmission	– Measles surveillance data (case-based and aggregate reporting systems)	– Surveillance sensitivity and specificity vary by time and place but often this is ignored in looking at trends	More systematic analysis of patterns of surveillance sensitivity using reported indicators (rate of laboratory investigation; rate of rash-and-fever cases that are not measles) shows this variation which can then be accounted for in trend analysis and compared to seroprevalence data if this is available

Planning future SIAs: when, where, and what age group?	– Birth rates and routine coverage used to estimate build-up of preschool-age susceptibles; follow-up SIA recommended when this equals one birth cohort	– Ignores susceptibility among older persons, which is now substantial in many countries – Ignores meta-populations	Models can estimate number of susceptibles each month/year, by space and age, using data on demography, coverage and incidence (adjusted for under-reporting). Through this, a model can predict impact of SIAs with various upper age limits at national or subnational levels to assess marginal benefits and cost. Models can also consider seasonality of measles and ability to implement SIA well to examine trade-offs in delaying an SIA in order to implement it better

How effective is outbreak response vaccination?	– Measles cases reported via case-based and/or aggregate reporting systems before and after outbreak response conducted	– Outbreak response often occurs at or after the peak in cases – difficult to know the impact on outbreak duration – Outbreaks often occur in areas with poor pre-existing surveillance (or may have truly had very low incidence), so little or no pre-outbreak data for comparison	Use birth rate, coverage and incidence data to estimate patterns of immunity and identify spatial regions and age ranges that would be of high priority for outbreak response. Ideally should continue laboratory investigation over the course of the outbreak (can get concurrent measles and rubella or other outbreaks of pathogens that cause rash and fever) and adjust estimates over time as initial outbreak data are reported

What is the effect of vaccination strategies on Congenital Rubella Syndrome (CRS) burden?	– CRS surveillance conducted at sentinel sites to monitor trends – Serological surveillance of antenatal clinic attenders to monitor trends in susceptibility	– Very few CRS surveillance sites – Very little pre-vaccination data – Sentinel sites not representative (most likely in high density population areas with good laboratory support, whereas small, remote populations are most at risk of an increase in adult susceptibility)	Models can project which areas will have the highest theoretical risk, allowing for targeted further investigation and action

## 2 Program questions, problems and model contributions

### 2.1 Where are the gaps in vaccine coverage and where are most unvaccinated children?

#### 2.1.1 Background – vaccination coverage estimates

Countries report administrative coverage for RI vaccination, estimated from the reported number of doses delivered divided by the size of the birth cohort, to WHO annually. Administrative estimates of SIA coverage are reported separately to WHO. Because administrative estimates are often of poor or unknown quality [3], [11], [12], Gavi recommends regular, high quality vaccination coverage surveys and now requires a high-quality post-campaign coverage survey (PCCS) after every SIA it funds [6].

#### 2.1.2 Problem – identifying gaps in vaccination coverage

Administrative coverage estimates suffer from inaccurate estimates of the target population (e.g. due to using outdated census data and failing to account for seasonal changes in the residential population [13]) as well as inaccurate counts of doses received (e.g. errors in tallying doses; doses administered to persons excluded from the denominator such as temporary migrants or individuals outside the target age group) [3]. Denominator problems are amplified in calculations of numbers of unvaccinated children, especially at small spatial scales. Surveys can potentially provide higher quality data, but due to costs, typically aim to measure coverage only at national or the first subnational level (e.g. province) and are infrequent [12].

#### 2.1.3 Model contribution

Geospatial modelling of data from high quality surveys that incorporate geographic coordinates of households allows estimation of coverage at a much finer spatial scale that is more actionable for program managers (Table 1). Models leverage the spatial autocorrelation in the data, as well as geospatial socio-economic, environmental, and physical covariate information from multiple other sources, to predict vaccination coverage at scales smaller than those for which the original survey was designed [14], [15], [16], [17]. This can highlight inequities in coverage which may be masked when analyses are done at provincial or district level [3], [15], [18] and can show contiguous areas of low coverage across countries [16], [17], [18]. Since model estimates at fine scales have wide uncertainty bounds [14], [15], they should be considered as highlighting areas where further investigation (e.g. local mini-censuses of vaccination status [19]), would be more fruitful. Fine scale coverage estimates can be integrated with corresponding geospatial model-based population estimates [20] to map the density of unvaccinated children [15].

Statistical modelling can identify cofactors that correlate with the geospatial distribution of low coverage (e.g. remoteness [15], [21], border areas with transient populations, and financial restrictions to health care [18]). Areas with the highest birth rates and lowest coverage are likely to be those with lower maternal educational levels [22] and socio-economic status [23], where measles case fatality may be highest, and are priorities for RI strengthening.

Administrative coverage estimates and many surveys only provide information on a single birth cohort (the most recent year for administrative data and often 12–23 month old children in surveys). It is important to consider coverage and immunity across all age cohorts, not just the most recent for which coverage is available. Statistical modelling also allows us to characterize the age-specific likelihood of vaccination [24], [25], informing estimates of the cumulative numbers of persons susceptible to measles (see next section).

### 2.2 How many people are susceptible to measles in different age groups, and where are they*?*

#### 2.2.1 Background – population susceptibility

The distribution of persons unvaccinated against measles is a useful indicator of program performance but an incomplete assessment of population immunity and its complement, susceptibility. Population immunity derives from vaccination (accounting for vaccine failure [26]) and natural infection. In some high birth rate settings, immunity via maternal antibodies in young infants, which may be lost before the age for routine MCV, is also important [27]. Therefore, estimates of population susceptibility must account for immunity from vaccination, natural infection, and passively acquired maternal antibodies.

#### 2.2.2 Problem – accounting for immunity from natural infection

Measles cases presenting to health facilities may be reported as aggregate numbers per week or month, and/or may be included in a separate case-based reporting (CBR) system [28]. CBR classifies cases as laboratory-confirmed if a blood sample is taken and the virus or measles-specific IgM antibodies are detected in an accredited laboratory; epidemiologically linked if the case is geographically and temporally linked to a laboratory-confirmed case, and clinically compatible if the clinical case definition was met but neither of the former two definitions apply. Countries report the number of measles cases to the WHO and UNICEF annually on the Joint Reporting Form [28], but the source of data (aggregate or CBR) and definitions followed vary over time and place, and reporting is insensitive. Worldwide, less than 3% of cases that models predict to occur [9] are reported [28].

Serological surveys conducted to measure measles sero-prevalence can be useful to show the distribution of immunity to measles, but they have many challenges including time, cost and varying assay sensitivity [7], [29] and only provide a cross-sectional snapshot of the immunity profile [30]. As an alternative to single-purpose surveys, countries can look for opportunities to include measurement of measles antibody in surveys whose primary objective might be different (e.g. HIV sero-surveys [31]). An innovative approach to obtain longitudinal data is to use residual serum samples from rash-fever surveillance programs, but how well these represent the population of interest needs assessment [32].

#### 2.2.3 Model contribution

Models can help address some of the limitations of vaccination coverage and disease surveillance data sources [8], [33], [34], [35], [36], in order to estimate population susceptibility. If data from serological surveys are available, they can be incorporated in a statistical model to estimate a current susceptibility profile from a past cross-sectional snapshot of immunity [37]. Statistical models are useful to examine variation in surveillance quality within and between countries and adjust surveillance data for under-reporting [38] and, where available, age-specific rates of laboratory confirmation.

**Fig. 2:**
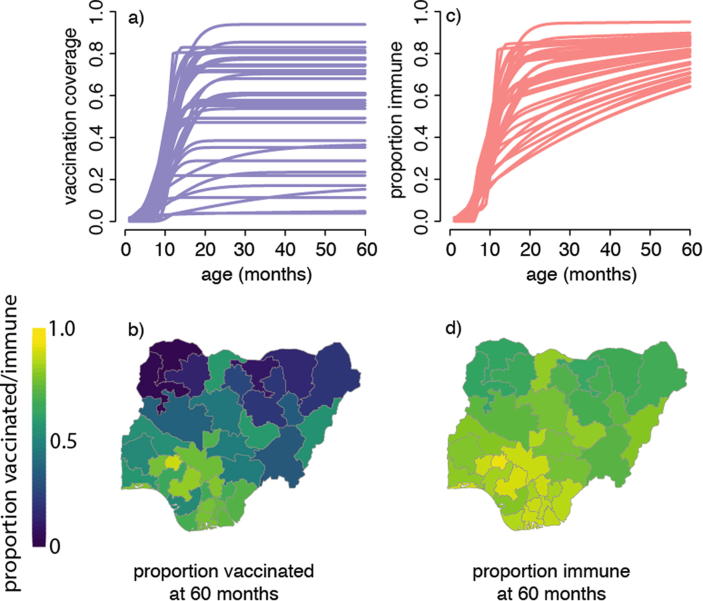
Illustrative comparison of the expected proportion vaccinated against measles and the expected proportion immune as a function of age. (a) The fitted proportion of children that have previously received measles vaccine (all sources, including RI and SIAs) at each month of age between 0 and 59 months per state in Nigeria, as estimated from the 2013 Demographic and Health Survey. All clusters within each state were combined and the proportion reflects both maternal recall and vaccination cards. (b) The proportion of children of age 60 months that have been previously vaccinated per state in Nigeria according to Nigeria's 2013 Demographic and Health Survey. (c) The expected proportion of children that are measles-immune at each month of age between 0 and 59 months per state in Nigeria in 2013, assuming vaccination coverage as in a) and a constant force of infection (FOI) in all states; the assumed FOI is consistent with a mean age of infection of 5 years in the absence of vaccination. (d) The expected proportion of children aged 60 months immune to measles (due to either prior vaccination or infection) per state in Nigeria in 2013; vaccination coverage and FOI assumption as in (c).

Estimated age-specific natural infection rates and historical data on RI coverage and SIAs can be used to calculate the proportion of the population in different age groups that is immune, and combined with regional birth rates to estimate the size of the annual susceptible birth cohort and thus the cumulative distribution of susceptible persons by age group at the desired spatial scale [16] as illustrated in Fig. 2. Susceptibility profiles do not necessarily match coverage profiles [30], [38] because in areas of low vaccination coverage, natural infection may generate a relatively large fraction of overall immunity, resulting in more homogeneous patterns of immunity than of vaccination coverage (Fig. 2a-d). Indirect protection due to herd-immunity implies that the force of infection, and thus the contribution of natural immunity to overall population immunity, will be relatively smaller in regions with higher vaccination coverage. Further, the absolute number of susceptibles depends on the interaction between population density and the distribution of immunity (see below), hence examining data on coverage alone is insufficient to predict risk of transmission.

### 2.3 How likely is measles transmission in different populations?

#### 2.3.1 Background and Problem – persistence and elimination of measles

Persistence of measles transmission in a population depends on the numbers and distribution of susceptible individuals, the rate at which they are replenished by births (minus the number immunized) [39], [40], migration of susceptible persons and infected individuals [41], and socio-behavioral factors affecting the chances of contact of a susceptible individual with an infectious individual [8], [42], [43]. Nonlinearities in transmission dynamics also make predicting risk more complex. Simple analyses of vaccination coverage and reported incidence trends at district level may lead to erroneous predictions of risk, due to suboptimal data quality, heterogeneities at finer spatial scales and contacts between wider populations (see below).

#### 2.3.2 Model contribution

**Fig. 3:**
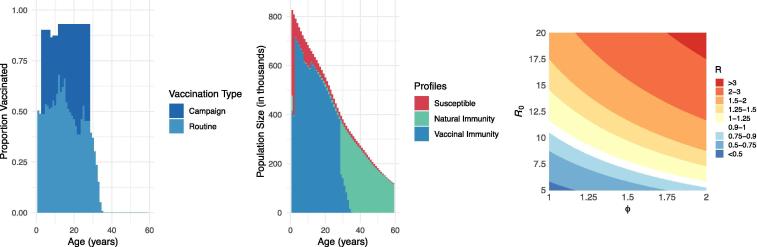
Benefits of modeling to understand outbreak risk. (A) From readily available data, we can derive the proportions vaccinated by age cohort; here we see the vaccination profile as of 2018 in Madagascar vaccinated by routine (WHO-UNICEF estimates) and campaign (administrative coverage reported to WHO) activities. It is important to note that administrative coverage data typically over-estimate campaign coverage, and a post-campaign coverage survey is preferred when available [12]. (B) To infer population susceptibility prior to the 2018–19 measles outbreak, we can use modeling to estimate age profiles of measles susceptibility, natural immunity, and vaccinal immunity; here we portray the epidemiologic profile of the Malagasy population estimated using pseudo-dynamic transmission models [16]. (C) Incorporating estimates of susceptibility heterogeneity, we can use modeling to estimate outbreak risk. Here we demonstrate the estimated measles R in the Malagasy population in 2018 across different assumed R_0_ values (5–20) and susceptibility clustering levels (defined as the relative probability of infected individuals coming into contact with susceptible individuals, e.g., *ϕ* = 1 in a homogeneously mixing population and *ϕ* = 2 when infected individuals are twice as likely to contact susceptible individuals) [47].

Models that integrate data describing characteristics of persistence can be used to estimate the spatio-temporal and age-specific dynamics of transmission risk. One simple yet critical metric of transmission risk is the effective reproductive number, R. This is defined as the average number of secondary infections produced by a typical infective person in a population, having accounted for existing population immunity via prior immunization or infection. Once an infection has been eliminated, R must be maintained below 1 to prevent the re-establishment of endemic transmission in the event of re-introduction [44]. The level of measles population immunity required to sustain R < 1 (the so-called “herd immunity threshold”) is generally estimated to be above 90% [45], [46] and as high as 96% when susceptibility and contact patterns cluster (see below) [47]. Dynamic transmission models offer a way to estimate R by integrating profiles of susceptibility with data on population demography, pathogen biology, and contact patterns that can then be used to estimate incidence and subsequently the likelihood of achieving elimination or experiencing an outbreak [48], [49] (Fig. 1, Fig. 3).

**Fig. 4:**
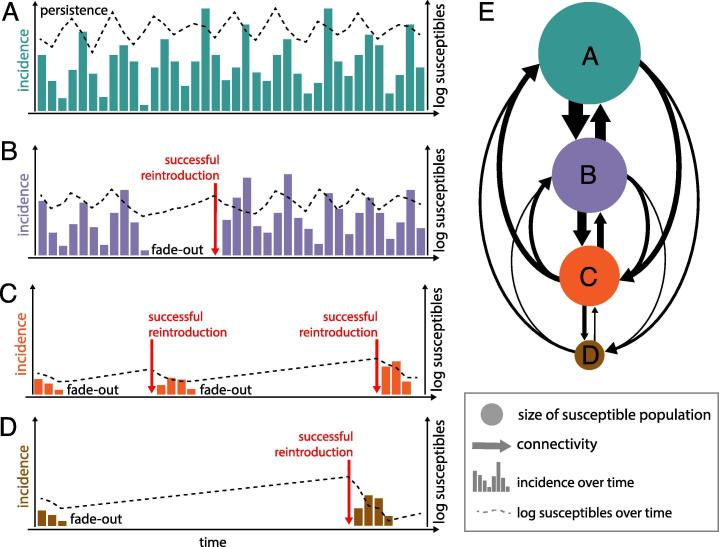
Schematic demonstrating effects of population size and connectivity on transmission dynamics. Number of incident cases (bars) and number of susceptible population (dotted line) in a (A) very large susceptible population that is highly connected, (B) large susceptible population with moderate connectivity, (C) medium-size susceptible population with lower connectivity and (D) in a small susceptible population with low connectivity. (E) The connectivity and susceptible population size of the four populations shown in A–D. Populations are connected to the other populations proportional to the thickness of the arrow and whose susceptible population size is proportional to the size of the dot. Persistence and fade-out are determined by the number of susceptible individuals in the population and contact between susceptible and infected individuals. Therefore, populations with more susceptible individuals experience persistence. After fade-out, successful re-introduction (represented by the red arrow) of infected individuals is determined by connectivity and the size of the susceptible pool. Larger susceptible pools in well-connected populations will likely experience shortened delay between fade-out and successful re-introduction. (For interpretation of the references to colour in this figure legend, the reader is referred to the web version of this article.)

Though the likelihood of eliminating measles is negatively correlated with R, the exact relationship is complicated and functionally unknown in most operational settings. Measles incidence may drop to zero even when R > 1 due to random chance if low absolute numbers of infectious cases fail to transmit before recovering. This phenomenon, referred to as stochastic fadeout, is more likely in small and remote communities [50]; thus, the absence of infection may not strictly indicate R < 1. Conversely, measles may persist regionally despite all, or many, communities maintaining R < 1 due to so-called “metapopulation dynamics” [51] (Fig. 4). If infected individuals travel, then measles may persist at the regional scale, despite only short, small outbreaks in any given location. The likelihood of persistence at the regional (metapopulation) scale depends on both the distribution of R at the local level (reflecting the degree of spatial aggregation of susceptibility) and the rate of introduction of infection into susceptible populations. Introductions depend on the degree of connectivity to areas where measles is circulating, for example, via main road networks between neighbouring districts and countries [52], [53], or through international travel [54], [55]. Since introduction events can occur on regional or national scales, broader regional dynamics should be considered to estimate the potential for achieving or sustaining elimination [52], [53], [56].

Patterns of contact between different population groups also influence transmission. Low coverage in groups with high contact rates may increase outbreak risk to a greater extent than previously appreciated [47], [57]. Heterogeneities in contact by age have been extensively demonstrated and these dynamics may interact with susceptibility profiles over space. Dynamic transmission models predict that vaccination should increase the mean age of infection and increase inter-annual variability in incidence [46], although this effect correlates inversely with birth rate [41]. Schoolchildren have high contact rates and represent a large proportion of the population in LMICS [58]. Once vaccination raises the mean age of susceptibility from pre-schoolers to school-age, the probability of transmission among susceptible persons may increase [58], although if there are many fewer susceptible people overall, incidence in the total population will decrease.

Seasonal changes in population demographics, contact patterns and access to vaccination should also be considered [32], [41], [52]. Major seasonal migration patterns (e.g. linked to agriculture [52], [53], school holidays [59], national holidays [60], or rural–urban migration [38], [56]) can drive measles fadeouts (when susceptible individuals leave) and outbreaks (where susceptible individuals accumulate and measles virus is imported into the community).

As measles becomes rare due to vaccination, these and other nuances of local conditions take on a bigger role in the dynamics of persistence. Dynamical transmission models are therefore useful in (1) exploring the relative contribution of different processes in persistence at low susceptibility (e.g. metapopulation dynamics, connectivity, clustering, age-specific transmissibility, rates of waning of maternal immunity), (2) identifying the empirical patterns that would indicate which processes are important in a given setting (e.g. changes in age distribution of cases), and (3) characterizing the relative benefit of alternative interventions conditional on the specific local context.

### 2.4 How effective are different strategies to reduce levels of population susceptibility and the chance of measles transmission?

#### 2.4.1 Background and Problem – routine vaccination with 1 and 2 doses of MCV

Since 2017, WHO and Gavi have encouraged all countries to schedule a second routine dose of MCV (MCV2), irrespective of the coverage of the first routine dose (MCV1), in order to provide an additional opportunity to reach those missed in infancy (in effect, a late 1st dose) and to immunize those who failed to respond to their first dose (a true 2nd dose) [2]. The focus on MCV2 introduction might shift attention from ensuring that infants receive MCV1, and there is potential confusion around reporting of doses administered at or after the scheduled age for MCV2 among children who missed MCV1 in infancy.

#### 2.4.2 Model contribution – quantifying burden outcomes of MCV2 as a late 1st dose vs. a true 2nd dose

The effect of MCV2 on population immunity depends on whether it is a true 1st or 2nd dose – which depends on the degree to which receiving MCV1 is correlated with attending for MCV2 [61]. If they are highly correlated, MCV2 is likely a true 2nd dose and the maximum change in immunity will be around 8% extra protection among vaccinated individuals (since vaccine effectiveness is around 92% in the 2nd year of life and 84% at 9 months [26]). This would be a useful step towards attaining the immunity levels needed for elimination in countries with very high MCV1 coverage. By contrast, when MCV1 coverage is low, an additional 8% immunity among twice-vaccinated individuals is a much smaller effect than that achievable by reducing the number of children who receive no MCV (so-called “zero-dose” children). Every opportunity should therefore be taken to vaccinate all children who missed one or both MCV routine doses at any health services contact, beyond the traditional 12 months of age [2].

The likelihood of receiving a second dose via an SIA may similarly be correlated with first dose if delivery is biased towards individuals with higher access to vaccination services. Dynamic models have been used to evaluate whether burden reductions following SIAs are consistent with the SIA dose being correlated with prior vaccination or not [38], [40]. A natural extension of these methods will be to evaluate whether the observed decline in incidence after introduction of a routine second dose is consistent with model predictions of delivery as a true second dose (small effect) or a first dose for zero dose children (larger effect).

An additional factor affecting impact on transmission is the age at each dose of vaccine, especially in high birth rate countries – the older the child is before receiving each recommended dose, the longer they are at risk of infection and contributing to the pool of susceptible persons. Improving monitoring of both the coverage and timeliness of each dose of vaccine is therefore important at national and subnational levels.

#### 2.4.3 Background and Problem – use of SIAs for measles control and elimination

SIAs are widely used to complement RI and can be a cost-effective measles control strategy [62]. They are, however, resource-intensive and can disrupt routine services, hence meriting careful evaluation. A variety of approaches are used to assess different potential indicators of success (Table 1).

**Fig. 5:**
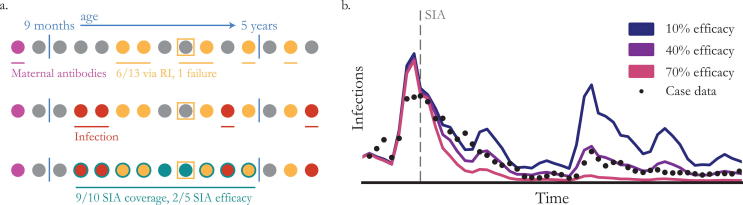
Estimating the impact of SIAs. (a) A hypothetical population of 15 individuals arranged by age (circles) illustrating the sources of measles immunity and their interplay. In the top row, maternal antibodies protect some of the youngest children (purple), while routine immunization covers 6 of the 13 eligible (yellow) with one vaccine failure (square). In the second row, natural infection affects 4 individuals across the age range (red). Finally, an SIA targeting the 10 individuals from 9 months to 5 years-old covers 9 (teal). Of the 5 individuals still susceptible (grey) at the time of the SIA, 2 are immunized in the campaign, implying that the SIA’s efficacy is 40%. (b) Dynamic models have been used to estimate SIA efficacy by computing goodness of fit to incidence data as SIA efficacy changes. In this illustrative example, 40% SIA efficacy (purple) does the best job of explaining measles incidence (black dots) after the SIA (grey dashed line). (For interpretation of the references to colour in this figure legend, the reader is referred to the web version of this article.)

SIA coverage is reported to WHO as the proportion of all persons in the target age group who received vaccine during the SIA; however, because of inaccuracies in denominators and numerators, administrative coverage reports are frequently over 100%. Well-conducted PCCS can provide more accurate data especially if done very soon after the campaign when finger mark or card evidence of vaccination may be available [63]. An important indicator of SIA effectiveness is the percentage of “zero-dose” children who are reached by the SIA – this should be explicitly measured through PCCS. However, the accuracy of information on previous vaccination is unknown, especially for older children whose vaccination records may have been lost [64] and who may have been eligible for inclusion in multiple previous campaigns. Furthermore, coverage of the target population does not equate with impact on susceptibility because targeted individuals may be immune before the SIA (Fig. 5).

If measles surveillance systems have consistent reporting rates over time, SIA impact can be inferred by decreases in reported incidence after an SIA [38], [65] although if measles incidence shows strong seasonality and an SIA occurs after a peak in incidence, susceptible individuals may have been depleted irrespective of the SIA [66]. Measles surveillance methods change over time [67] and in areas with weaker health service infrastructure, where SIAs would be expected to have the greatest effect, reporting may not be sensitive enough to detect changes.

Deciding on the timing and scope (age range and geography) of future SIAs is also challenging (Table 1). In most Gavi-eligible countries, current strategies are not predicted to keep R < 1 [68], hence outbreaks will continue to occur and their timing will become less predictable as birth rates decrease and coverage increases [4]; optimizing outbreak response vaccination (ORV) is an additional challenge.

#### 2.4.4 Model contribution – evaluating SIA success

Geospatial analyses of the coverage of the trivalent Diphtheria, Tetanus, Pertussis vaccine (DTP) (given via RI) can be compared to those of MCV1 (given via RI and SIAs). Where MCV1 coverage is much higher than DTP coverage, the SIA may have been effective, although other reasons for the differences cannot be excluded [69]. Model-based estimates of denominators [70] can be used to improve estimates of target populations for SIAs and can be integrated with estimated coverage before and after the SIA to assess the reduction in the number of unvaccinated individuals.

Since most individuals targeted for SIAs were immune before the SIA due to previous vaccination or infection, the difference in population immunity post-SIA compared to pre-SIA is the strongest evidence of success (Fig. 5). Only a few studies have measured this directly [71], [72], [73], but models can estimate the contribution of SIAs to population immunity [39], especially if serological data [74], [75], [76] or high quality case-based incidence data [38], [40], [77] are available post-SIA*.*

#### 2.4.5 Model contribution – deciding when and where to conduct SIAs

The impact of SIAs depends on the ability to identify immunity gaps accurately by age and space, and to reach the individuals targeted. Models can examine the trade-offs between the degree to which an SIA would theoretically increase population immunity and the ability to reach different age groups effectively. Prada et al. [68] predict that, in a large country with high birth rates and low routine coverage, a well-conducted SIA targeting children under five years will not entirely prevent outbreaks (since some older persons are susceptible) but is more cost-efficient than a less well-implemented SIA targeting a broader age group. Increasing the upper age beyond age 10 years would have small marginal benefit even if implemented to the same standard. By contrast, after high routine coverage is sustained for many years, the age pattern of immunity changes [39], [78]. As countries progress towards elimination, the upper age that an SIA would have to target to reach 90% of the remaining susceptible individuals increases [4]. In many LMICS (and most high-income countries) susceptible adults would need to be vaccinated to achieve population immunity targets of at least 95% [4], [39].

When seasonality of measles incidence is strong, vaccinating when susceptible build-up is high, but before the onset of the high-transmission season, is predicted to have greatest impact [79], but the incidence of measles in previous years also needs to be accounted for [38]. Ease of access to vaccinate population groups may also vary seasonally, hence SIAs could be timed when the greatest numbers are in areas considered easier to reach [80], [81], [82]. Some large countries like the Demographic Republic of the Congo and India stagger implementation of SIAs over 2–3 years. If the lowest coverage areas are left until last, high burden will continue for longer pre-SIA, but giving those areas more time to prepare might enable the SIA to more effectively reach zero-dose children, and assuming the campaign pushes populations into synchronous local elimination, it optimizes the potential for country-level elimination.

#### 2.4.6 Model contribution – duration of SIA impact

The duration of impact depends on the rate at which susceptible persons accumulate after the SIA; duration is shorter when birth rate is high and/or routine vaccination coverage is low [68], [83]. The WHO encourages countries to use available high quality data on birth rate, vaccination coverage, surveillance, and serological studies to monitor the accumulation of susceptible people and conduct follow-up campaigns before the number of pre-school children susceptible to measles approaches the equivalent of one birth cohort, in order to prevent a measles outbreak [2]. This rule of thumb ignores the contribution to outbreak risk from older persons who are susceptible (see above), susceptibility among infants before the age for routine MCV1, heterogeneity in vaccination coverage, and connectivity within meta-populations, all of which may make outbreaks more likely sooner than predicted from average national data. In countries that exhibit strong internal heterogeneities in RI and birth rates, total susceptibility and susceptible density will accumulate heterogeneously, and models predict that tailoring the inter-SIA period to conditions at sub-national scales can potentially be more cost-effective and equitable than periodic nationwide campaigns [84].

#### 2.4.7 Model contribution – outbreak response vaccination

Models help prioritize areas for intervention using different metrics of risk [85] and predict the impact of different ORV strategies. Minetti et al. [86] estimated that making extra effort to vaccinate persons in the communities considered hardest-to-reach will have greater impact than conventional non-selective SIAs, which target all areas equally and may preferentially reach those already vaccinated [87].

When deciding on the scope of ORV, the age distribution of early cases often drives decisions [77]. Erroneous conclusions can be drawn early in the outbreak, however, due to biases in data on coverage and incidence [30], [88], [89]. If feasible, responses should be reconsidered and adapted as new data become available (e.g. updated incidence data with laboratory confirmation) [89], [90]. Constraints on the data available to inform decisions about the geographic scope of ORV may be even greater, as current methods to measure mobility are imperfect [88].

### 2.5 What populations are of special concern for rubella?

#### 2.5.1 Background and Problem

Since infection among women of childbearing age is of most concern, vaccination-driven dynamic changes in epidemiology result in unique considerations for control of this pathogen. Childhood rubella vaccination below a critical threshold of vaccination coverage can potentially result in a short-term or annual increase in the number of infants born with congenital rubella syndrome (CRS) by increasing the average age of infection without sufficiently reducing rubella incidence, an outcome reported in Greece and Costa Rica [91], [92], [93].

#### 2.5.2 Model contribution

Transmission models can be used to project the potential country-level impact of different rubella vaccination rollout strategies on CRS incident cases in the short [74] and long term [48]. Based on what is known about rubella transmissibility, the current strategy of a SIA targeting children up to age 15 years at the time of introduction of rubella vaccine into the routine program is considered unlikely to lead to a long-term (30 year) increase in CRS in LMICs as long as coverage is above 80% [48], however there remains risk of short-term increases in CRS [74].

At smaller spatial scales, local elimination and re-introduction dynamics of rubella can nonetheless result in older age profiles of infection in endemic settings among populations below the critical community size (CCS) [50], [94], [95], i.e., where rubella is prone to stochastic elimination. Isolated communities below the CCS, and where local elimination has occurred, may then experience many years of build-up of susceptible individuals (predominantly via births) before infection is re-introduced (Fig. 4). The result may be older ages of infection, and therefore a higher rate of CRS cases. Spatial heterogeneity in disease burden can be exacerbated once vaccination is introduced, if there is a strong correlation between population size and vaccination coverage [95], as a result of lower rates of re-introduction [25]. Models have been used to demonstrate these dynamics, and can be used, in combination with data on spatial demography and connectivity, to highlight populations at theoretical risk of higher burden of CRS post-vaccine introduction. Monitoring rubella epidemiology would be especially important in those areas [96].

## 3 Discussion

The demographic contexts and the effectiveness of vaccination program implementation vary greatly between regions and countries leading to a range of patterns of measles transmission [4], [66], and countries are now encouraged to carefully review and analyse data to adapt strategies according to the local situation [2], [6]. Models are useful tools to highlight gaps in program implementation and limitations of empirical data and to predict the effects of potential interventions.

Geospatial analyses can identify clusters of low coverage that often cross administrative or national borders [16], [17], [18], [97]; this should lead to investigation to confirm low coverage in the areas identified by models, determine the causes and stimulate appropriate action. Dynamic transmission and statistical models offer the advantages of combining multiple sources of data (e.g. demographics, connectivity, geospatial, coverage, surveillance, serology) to improve estimates of different indicators of program success and their interpretation [98]. Results suggest that focusing on a uniform percentage coverage goal across all districts may not be the most effective way to control measles – consideration of the absolute number of people susceptible to infection, and ideally also assessing contact patterns between them, may lead to different prioritization of areas for intensive efforts [99].

Results of dynamic models show the need to manage expectations about the predicted impact of currently supported interventions. SIAs targeting only children <5 years will not always avoid outbreaks because many countries have substantial immunity gaps in older persons among whom contact rates may be higher. Conversely, when very high routine coverage has been sustained, a shift from SIAs to more operationally challenging, targeted strategies such as selectively vaccinating only individuals without evidence of two prior doses may be more cost-effective [100]. In some countries, this might be achievable through school-entry checks.

By combining information from different data sources, models can help to address some of the biases in the original data which, if ignored, would often lead to erroneous conclusions [98], [101]. Models help to promote the use of more appropriate indicators of success, for example the effectiveness of SIAs in reaching “zero-dose” children and in reducing the number of persons susceptible to infection, rather than focusing only on the proportion of the entire target population vaccinated during the SIA (which is currently the main objective of PCCS [102]). It may be difficult to measure coverage precisely among “zero-dose” children in a PCCS because this would require prohibitively large sample sizes in countries with high routine vaccination coverage. Furthermore, “zero-dose” children may be more likely to be missed during household surveys as they may not be included in typical household listing procedures (e.g. homeless or highly mobile persons), be absent at the time of interview, or refuse to participate due to mistrust of authority. By including other sources of data such as disease incidence data, models can estimate the contribution of SIAs to population immunity and help to overcome drawbacks of single sources such as PCCS. Models have recently helped guide strategies at country level in Nigeria [103], Pakistan [38] and Madagascar [104].

In addition to helping address near-term programmatic questions, models are useful to investigate future trends, including the importance of susceptibility before age 9 months which may increase as the prevalence of natural immunity among women of childbearing age declines [105], [106], and the potential need for additional interventions to reduce susceptibility in older children and adults in the future.

Of course, modelling has its own unique challenges and limitations, including many not discussed in this paper, such as accounting for logistics and economics of vaccine delivery or immunodynamics [107]. Models are only as useful as the data that they are fit to and the assumptions made*.* To make better evidence-based decisions, more should be invested to improve empirical data. Geospatial analyses are constrained by the time before datasets are available (e.g. Demographic and Health Survey data typically refer to birth cohorts from at least 2 years prior to data release), infrequency and inconsistent quality of surveys; reliance on a verbal history [12] which may be less accurate for the number of doses received or source of vaccination [108], and wide uncertainty limits around estimates in sparsely populated areas [14], [69]. PCCS can give up-to-date information on SIA coverage, and planning for geospatial analysis should become part of their design, but they are still constrained by the quality of recall, especially for wide age ranges, and by potential selection bias as outlined earlier. Investment in strengthening administrative data systems, including censuses and birth registries as well as vaccination recording [109], is therefore important [110].

Measles and rubella surveillance need substantial investment – current rash and fever surveillance depends greatly on the infrastructure of the polio eradication program which is being wound down in many countries. Further improvements are essential to overcome disparities in reporting efficiency and laboratory investigation [111]. The use of serological surveillance in selected sites could be considered to monitor predicted changes in age-specific susceptibility [7]. Continued work is also needed to obtain and incorporate better data on mixing [112], connectivity and migration [20], [53], [59], seasonality [13], [52], [82], and demographic estimates from a range of spatial scales [70], [113]. Differences in contact rates by age has led to age-specific targets for prevalence of immunity in Europe [44] and further information on this is needed in developing countries. In addition, better reporting of deaths from measles and rubella could further improve program targeting to areas of highest mortality.

To move to a setting where planning of interventions is reactive to empirical data requires developing modelling infrastructure now, ideally through training of in-country partners [89], and improving communication about the uses and limitations of modelling [114]. Closer liaison between program managers, donors and modelers should be fostered. Much recent modelling has focused on planning and evaluating SIAs, but results should be used more to inform RI and the equity agenda. The more high-quality background data that are available, and the more pre-existing analyses and modelling done and effectively communicated, the better prepared a country and its international partners can be to conduct analyses of new outbreaks in real time and determine the best response (e.g. current work in Madagascar [104]). Measles surprises even the best-performing countries and a foundation for data-driven response needs to be established worldwide.

## Declaration of Competing Interest

The authors declare that they have no known competing financial interests or personal relationships that could have appeared to influence the work reported in this paper.
